# Faculty Members’ Perspective on Virtual Interviews for Medical Residency Matching during the COVID-19 Crisis: A National Survey

**DOI:** 10.3390/healthcare10010016

**Published:** 2021-12-22

**Authors:** Fadi Aljamaan, Fadiah Alkhattabi, Ayman Al-Eyadhy, Ali Alhaboob, Nasser S. Alharbi, Adi Alherbish, Badr Almosned, Mohammed Alobaylan, Hayfa Alabdulkarim, Amr Jamal, Sami A. Alhaider, Basim Alsaywid, Fahad A. Bashiri, Mazin Barry, Jaffar A. Al-Tawfiq, Khalid Alhasan, Mohamad-Hani Temsah

**Affiliations:** 1 College of Medicine, King Saud University, Riyadh 11362, Saudi Arabia; faljamaan@ksu.edu.sa (F.A.); aleyadhy@ksu.edu.sa (A.A.-E.); drhbooob@gmail.com (A.A.); nsalharbi@ksu.edu.sa (N.S.A.); aalherbish@ksu.edu.sa (A.A.); amrjamal@ksu.edu.sa (A.J.); fbashiri@ksu.edu.sa (F.A.B.); mbarry@ksu.edu.sa (M.B.); kalhasan@ksu.edu.sa (K.A.); 2 Critical Care Department, King Saud University Medical City, Riyadh 11362, Saudi Arabia; 3 King Faisal Specialist Hospital and Research Center, Riyadh 11211, Saudi Arabia; FKhatabi@kfshrc.edu.sa; 4 Pediatric Department, King Saud University Medical City, Riyadh 11362, Saudi Arabia; balmosned@ksu.edu.sa (B.A.); Mohammed.alobaylan@gmail.com (M.A.); hayfa.alabdulkarim@gmail.com (H.A.); 5 Department of Family and Community Medicine, King Saud University Medical City, Riyadh 11362, Saudi Arabia; 6 Evidence-Based Health Care & Knowledge Translation Research, King Saud University, Riyadh 11362, Saudi Arabia; 7 Saudi Commission for Health Specialties, Riyadh 11614, Saudi Arabia; s.alhaidar@scfhs.org (S.A.A.); drbasim@yahoo.com (B.A.); 8 Division of Infectious Diseases, Department of Internal Medicine, King Saud University Medical City, Riyadh 11362, Saudi Arabia; 9 Specialty Internal Medicine and Quality Department, Johns Hopkins Aramco Healthcare, Dhahran 34465, Saudi Arabia; jaltawfi@yahoo.com; 10 Infectious Disease Division, Department of Medicine, School of Medicine, Indiana University, Indianapolis, IN 46202, USA; 11 Infectious Disease Division, Department of Medicine, School of Medicine, Johns Hopkins University, Baltimore, MD 21218, USA; 12 Prince Abdullah Ben Khaled Coeliac Disease, Faculty of Medicine, King Saud University, Riyadh 11362, Saudi Arabia

**Keywords:** COVID-19, medical residency, virtual interviews, satisfaction, survey, faculty member

## Abstract

During the COVID-19 pandemic, conducting face-to-face medical residency interviews was challenging due to infection prevention precautions, social distancing, and travel restrictions. Virtual interviews were implemented by the Saudi Commission for Health Specialties (SCFHS) as an alternative process for residency matching while striving to maintain the same quality standards. This national survey was conducted to assess the satisfaction and perceptions of faculty members’ virtual interview performance in the assessment for the medical training residency programs. Among the participating 173 faculty members, 34.1% did not have previous experience with video-conferencing. The Zoom application was the most commonly used platform (65.9%). Most (89.6%) of the faculty perceived virtual interviews as “adequate” platforms on which the candidates could express themselves, while almost half of the faculty (53.8%) agreed that virtual interviews allowed them to accurately reach an impression about the candidates. Overall, 73.4% of faculty felt comfortable ranking the virtually interviewed candidates. We conclude that the acceptance of participating faculty members in the first Saudi medical residency training matching cycle virtual interviewing event was well-perceived. This study provides evidence for future application and research of virtual interviews in residency candidates’ assessment, especially after the pandemic crisis resolves.

## 1. Background

The application process for medical training programs consists of a series of steps that culminate with a personal interview. This interview is usually the last step before securing the slot. Data from human resources departments show that once candidates are selected for interviews based on their curricula vitae or application forms, they are mostly competing at the same level [1,2]. Interviews yield information about applicants that is not extracted from their resumes or personal statements and are also a chance for the interviewee to explore the institution. Interviews aim primarily to assess the candidates’ communication skills, in addition to exploring their aptitude, facial expressions, fluency, and certain clinical and ethical skills. Finally, interviews allow the assessment of the candidate for any red flags that would hinder their acceptance.

Program directors have reported that interviews are a key step in selecting candidates [3,4]. On the other hand, interviews have been criticized for being biased due to failure of standardization in many instances and low interviewer reliability and reproducibility [5,6,7].

Interviews have been traditionally conducted through the classical on-site face-to-face method. This method is popular owing to the advantages and intangible benefits of such interactions. However, this classic way of interviewing candidates has been criticized for multiple flaws, including the high cost for both parties [8], unfair competition among candidates who may be financially challenged [9], and limited interviewing slots causing scheduling conflicts and clinical work disruption [10]. As technology is one of the main enhancers of modern healthcare systems, adopting new trends in technology is crucial to optimizing their utilization in healthcare services and medical training [11,12,13]. Therefore, there has been a trend in the last decade towards video conferencing interviews.

Due to the COVID-19 pandemic, caused by the severe acute respiratory syndrome coronavirus 2 (SARS-CoV-2), traveling restrictions and infection prevention precautions, including social distancing and prolonged lockdown, were implemented in the Kingdom of Saudi Arabia. This situation made residency and fellowship training interviews challenging, which prompted exploring new dimensions in medical training interviewing and teaching [14,15]. The Saudi Commission for Health Specialties (SCFHS), the national regulator of postgraduate medical training in Saudi Arabia, decided to substitute on-site face-to-face interviews with virtual interviews in compliance with the different measures that emerged during the pandemic. This was especially important because both faculty members and applicants are healthcare workers who may pose a potential risk of spreading SARS-CoV-2 to different health institutions.

Several studies have evaluated virtual interviews in medical and surgical training programs, but most were individualized for one program and conducted in a single center. In addition, the virtual interviews were offered as an option or adjunct to the traditional face-to-face interview [16,17]. Most studies have concluded that virtual interviews can be a cost-effective and efficient alternative to on-site in-person interviews [8,16,17,18]. Nevertheless, being a newly introduced method, it has faced criticism and questions regarding its validity and acceptability by both applicants and faculty members. There is a growing need for studies to evaluate the performance of the virtual interviewing system and its implementation in medical residency programs, keeping in mind what has been said regarding its importance in the final selection of trainees and its consequences. Thus, this study was conducted to assess different aspects of faculty members’ satisfaction, acceptance, and perception of the performance of the virtual interview system that has been used by SCFHS for the matching cycle of March 2020 during the COVID-19 pandemic.

## 2. Materials and Methods

This is a cross-sectional study using a self-reported survey that was sent through email invitation to all faculty members who participated in virtual interviews during the March 2020 SCFHS matching cycle for the residency programs. The survey assessed their satisfaction and perception of the virtual interview performance from different domains, including applicant evaluation, audiovisual quality, applications used, preparation, event-associated stress, and others using Likert scales (Appendix A).

## 3. Sampling Technique

A non-probability, consecutive sampling technique was implemented. An invitation to participate in the survey was sent to all faculty members (200 members) who participated in virtual interviews for the matching cycle of March 2020. The response rate of the invitees was 86%.

## 4. Data Analysis

The means and standard deviations (SDs) were used to describe continuous variables, and categorical variables were presented as frequencies and percentages. The statistical normality assumption was examined using histograms and the Kolmogorov–Smirnov statistical test, and the statistical equality of the variance assumption was assessed using the Levene’s test.

Cronbach’s alpha test of reliability was performed to assess the internal consistency of the Likert-based items characterizing different concepts. In addition, a multiple-response dichotomy analysis was performed to describe the frequencies and percentages of the tick-all-that-applies questions. The overall mean scores of the Perceived Stress Scale and Perceived Satisfaction Scale with video-conferencing were computed by adding the items comprising these concepts and dividing the sum by the number of items for each concept. Statistical Package for the Social Sciences (version 21; IBM Corp., Armonk, NY, USA) was used for all statistical data analyses, and *p*-values of less than 0.050 denoted statistical significance.

## 5. Data Collection

The survey tool was developed based on a related literature review by an expert panel to reflect the participants’ previous experience, previous exposure to virtual platforms and their types, their perception of the current virtual interview performance, their satisfaction with different aspects of the virtual event, and their willingness to be involved in the virtual interviewing process in the future. A pilot study involving 10 faculty members was conducted to validate the tool for clarity and consistency. The Cronbach’s alpha test of reliability for the five0item Likert scale was 0.807 and 0.787 for the 10-item Likert scale, indicating reliable valid internal consistency. Then, the questionnaires were sent electronically to the participants within four weeks of the virtual interviews (15–30 April 2020), with reminders for non-responders after two days [19].

## 6. Ethical Approval

This study on the rapid utilization of video-conference interviews in the residency application process was conducted in accordance with the postgraduate center’s recommendations (memo #9/3/311082). This study had the approval of the SCFHS’s Institutional Review Board (#0420-03 exp). Data were collected after the participants had provided their informed consent. Participation was voluntary and not linked to the applicants’ evaluation. The results of this study will be used as a quality improvement project of the SCFHS.

## 7. Results

**Figure 1:**
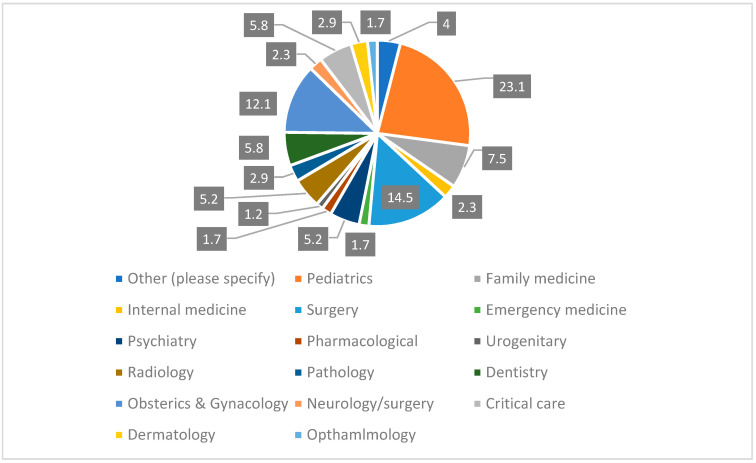
Faculty members specialty (in %).

A total of 173 faculty members completed the survey. Of these, 23.1% were pediatricians, 14.5% were surgeons, and 12.1% were from obstetrics and gynecology. The other specialties’ contributions ranged between 1.2% and 5.8% (Figure 1).

The members were split equally in their years of practice as consultant/faculty members: less than 5 years, more than 10 years, and between 5 and 10 years. Regarding the results of their previous experience with video conferencing, 63% said they had used it but not for residency interviews, while 2.9% had used it for residency interviews, and 34.1% had no previous video conferencing experience at all (Table 1).

**Figure 2:**
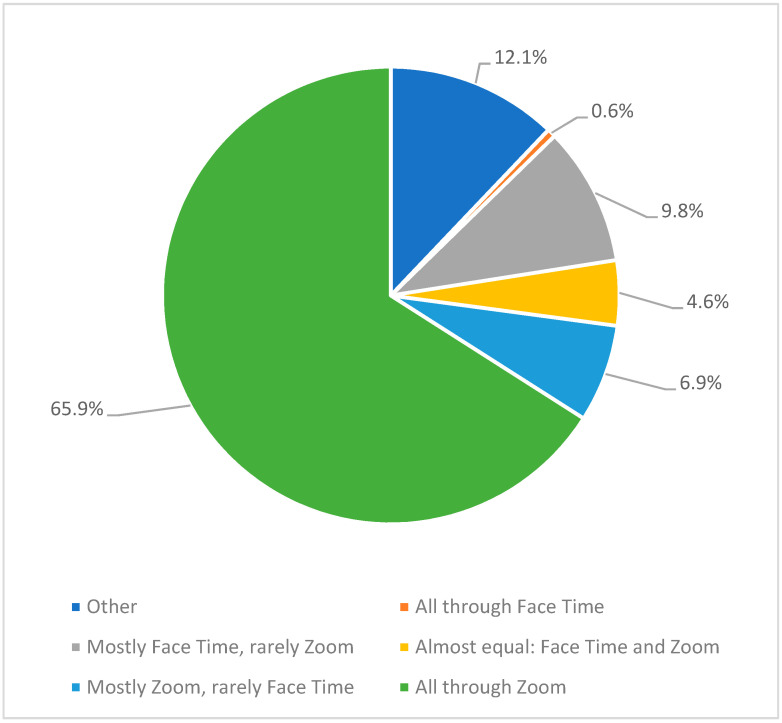
Video conferencing tools used by faculty members.

The faculty members were given the option to use multiple video conferencing software programs during the virtual interviewing event, including Facetime, Zoom, or another interface. Most of the interviews were entirely on Zoom (65.9%), 9.8% used mostly Facetime and rarely Zoom, and 0.6% used Facetime for all the interviews (Figure 2).

**Figure 3:**
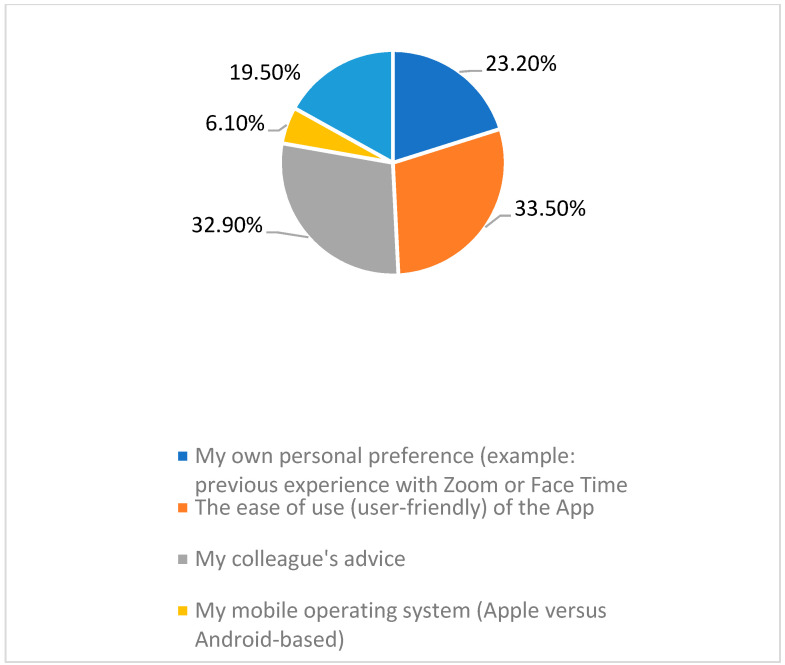
Faculty members’ motive of video conferencing tools choice.

About 55% of the faculty members chose the video conferencing software based on their personal preference and for its user-friendliness, while 32.9% chose it based on a colleagues’ advice (Figure 3).

Regarding the faculty members’ perception of the virtual interview performance, 89.6% agreed or strongly agreed that virtual interviews were adequate for the candidates to properly answer questions and express themselves, while 5.2% were neutral, and the remaining participants did not think they were adequate. On the other hand, 65.4% agreed or strongly agreed that the virtual interviews allowed them to accurately form an impression about the applicant’s personality, while 17.9% were neutral, and 16.8% were on the negative side.

Overall, most of the faculty members felt comfortable ranking the interviewed candidates based on the virtual interviews (73.4%), while 14.4% disagreed or strongly disagreed.

Overall, participating faculty members had high satisfaction with the virtual interview experience and would recommend its future use, with a mean rating of 8.37/10 (1.50) (SD). Regarding the faculty members’ perception of the interview’s organization and performance, 87.3% felt all or most needed information was given pre-event, while 10.4% felt only some needed information was given. With regard to the event’s organization, the majority (88.3%) felt that it was extremely or very organized, while 10.4% felt that it was only somewhat organized. As for the duration of the interviews, the majority (86.7%) felt that it was just the right duration (Table 2).

**Figure 4:**
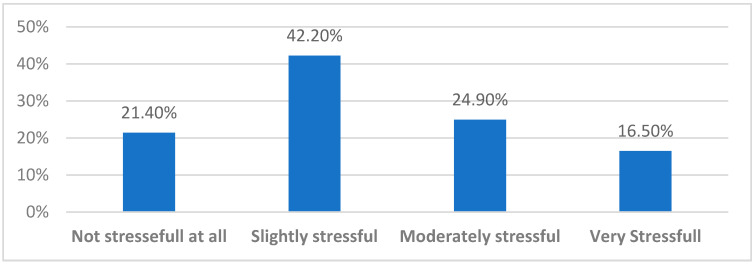
Stress level perceived by the faculty members.

Considering the stress level perceived by faculty members during the virtual interviews, 42.2% felt only slightly stressed, 24.9% felt moderately stressed, 21.4% did not feel any stress at all, and only 16.5% felt very stressed (Figure 4).

**Figure 5:**
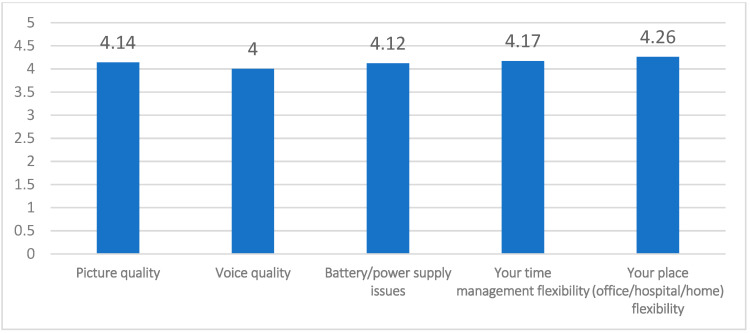
Faculty members’ (mean) satisfaction rate with virtual interviews domains of performance (maximum 5).

Regarding their satisfaction with the virtual interview, video, voice quality, time, and place flexibility all scored higher than 4 out of 5 (Figure 5).

**Figure 6:**
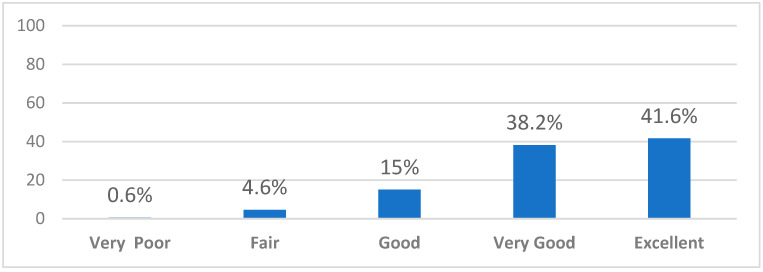
Faculty members overall rating of the virtual interview experience.

Their overall rating for the event was 4.16/5 (SD 0.89); 41.6% felt that the event was excellent, and 38.2% felt it was very good, while only 0.6% felt it was poor (Figure 6).

**Figure 7:**
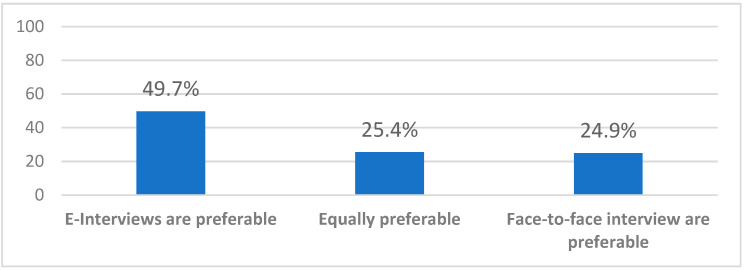
Faculty members future preference of virtual interviews vs. face-to-face.

The faculty members showed a high likelihood to recommend virtual interviews to their colleagues (8.37/10 SD 1.50 on Likert scale), but when asked about their preference to use them versus face-to-face interviews in the future, 49.7% preferred virtual interviews, 24.9% preferred face-to-face, and the remaining were neutral (Figure 7).

**Figure 8:**
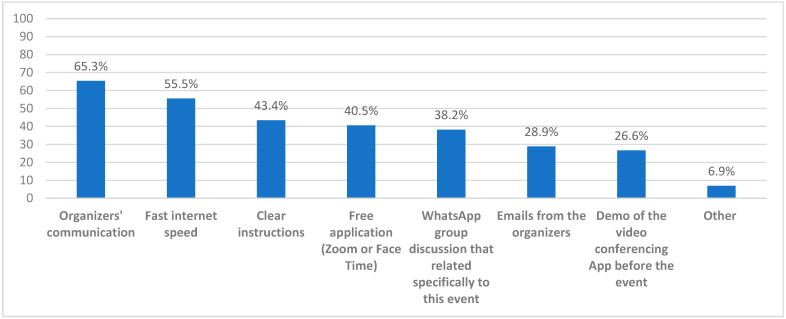
The faculty members’ perceived factors that enhanced the medical residents’ E-interviews experience.

We also surveyed the faculty members for the different factors they perceived may have positively or negatively affected the residents’ virtual interview experience. The majority (65.3%) believed that organizers’ communication was the most important enhancer, followed by optimal internet connection speed (55.5%), clear instructions (43.4%), and the video conferencing software being free of charge (40.5%) (Figure 8).

**Figure 9:**
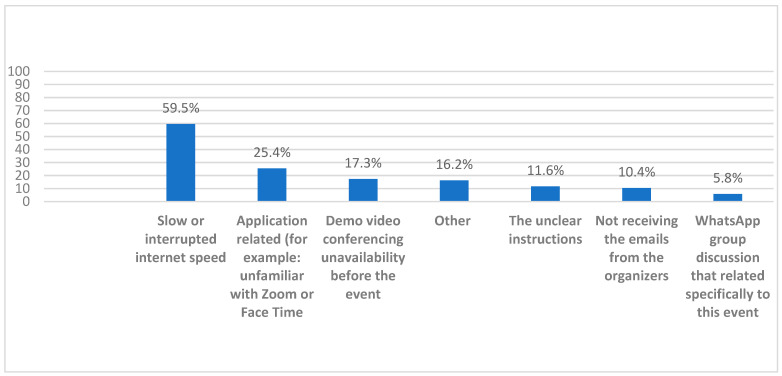
The faculty members’ perceived factors that negatively affected the medical residents’ E-interviews experience.

Regarding the negative impactors, 59.5% said that internet connection speed was occasionally slow or was interrupted and that might have negatively affected the residents’ experience, 25.4% felt that applicants were unfamiliar with video conferencing software, and 17.3% expressed that the lack of a demonstration video on how to use the tool prior to the event was a major negative impactor. For other enhancers and negative impactors, see Figure 9.

## 8. Discussion

Virtual interviews have been used for almost a decade in the medical residency and fellowship matching process. Many studies have been published worldwide to assess their performance and faculty and applicants’ satisfaction with them; however, they have not been introduced in the matching system in Saudi Arabia before. When the COVID-19 pandemic affected Saudi Arabia in March 2020, different mitigation measures were implemented, including lockdowns, prevention of gatherings, and travel restrictions [20,21,22]. These unique circumstances prompted the SCHS to introduce virtual interviews for the March 2020 cycle.

Our study aimed to survey the faculty members’ experience with Saudi’s first virtual medical residency interviews. Our results have shown high faculty satisfaction with this experience, which matched the international literature, even though the latter has been inconsistent in its results to some extent [23]. A recent Mayo Clinic study during the COVID-19 pandemic showed high satisfaction of program directors (PD) with virtual interviews (mean 4.65/5), which is similar to our result for all faculty members (4.16/5); however, in their study, just over half of all PD agreed or strongly agreed that next year’s interviews should be virtual regardless of the COVID-19 status [24]. Another study conducted during the COVID-19 pandemic addressed neurosurgery residency program virtual interviews and showed that 57% of faculty agreed to implement virtual interviews in the future, while 44.5% expressed that they would not replace in-person interviews with virtual interviews in the future [25]. Both studies echoed our results as only 49.7% of the faculty members preferred virtual interviews in the future, and 24.9% preferred face-to-face interviews (Figure 6). Another study during the pandemic that evaluated the cardiothoracic match for the year 2020 has shown reasonable PD satisfaction with virtual interviews, as 55% of them agreed or strongly agreed that they should be offered in the future, while only 15% of them agreed or strongly agreed that it should be offered without the option of an in-person interview [23]. Thus, so far, most evidence supports that faculty members and program directors have shown good satisfaction with virtual interviews but are still not fully confident of replacing face-to-face interviews with virtual interviews in the future. One study explored the lack of trust in virtual interviews; it showed that 50% of PD suggested that virtual interviews made the selection committee rely much more on the applicant’s objective data rather than the interview itself; 75% and 67%, respectively, disagreed or strongly disagreed that E-interviews allowed easier assessment of the applicant’s fit, personality, and communication skills [26]. In contrast, our study was highly encouraging from the faculty point of view, as 65.4% of the faculty members agreed or strongly agreed that virtual interviews allowed them to form an impression about the applicants’ personality, while 17.9% were neutral, and 16.8% were unsatisfied. Additionally, 73.4% felt comfortable ranking candidates based on their perceived impression, and 89.6% agreed or strongly agreed that virtual interviews were adequate for candidates to answer different questions and properly express themselves. In comparison, a recent local study that assessed applicant’s satisfaction with virtual interviews showed that only 37.3% and 14.9% agreed or strongly agreed, respectively, that the interview allowed them to accurately represent themselves, while 63.3% would prefer virtual interviews over face-to-face interviews in the future [27].

In our study, the faculty members had a variable amount of trust in some assessment domains regarding the virtual interviews. This may be magnified when we compare the faculty’s high satisfaction with the virtual interview performance of almost 90%, while only 73.4% felt comfortable ranking the interviewed candidates based on these interviews. On the contrary, only 53.8% agreed that the virtual interviews allowed them to assess the applicant’s personality accurately. Although this may seem contradictory to the results, it may point to certain defects in the performance or the assessment process that our study, similar to others, could not examine further, which culminated with the faculty perception when asked about their future preference between virtual interviews and traditional face-to-face interviews; 49.7% preferred the virtual interviews, while 24.9% preferred face-to-face interviews.

The majority of faculty members (63%) had used video conferencing tools previously, especially because of the current COVID-19 pandemic and the wide applicability of such tools for administrative meetings, webinars, and clinical-based meetings; this created familiarity with digital technology and its healthcare and medical education applications. This was reflected clearly in their low stress level, as about 80% felt slightly stressed or not stressed at all by the virtual interview process.

Virtual interview organization, telecommunication quality, and the interview setting’s flexibility are crucial items to be addressed when introducing digital video conferencing in the residency matching process. Our literature review revealed a lack of data pertaining to the performance domains of the virtual interviews. One of the strengths of our study is that we explored the faculty members’ perception of the interview performance regarding their quality indicators from different points and their flexibility for usage in terms of place and time, which showed decent satisfaction results [28,29].

Such virtual experiences, being newly introduced worldwide and especially here in Saudi Arabia, need to be fortified and developed each matching cycle. Areas of improvement could be introducing virtual tours in training centers and didactic training courses highlighting the benefits and challenges of each residency program, helping the applicants to focus their decision compass.

Virtual interviewing is the future of the medical training matching process; it started before the current COVID-19 pandemic and has progressed quickly since then. Satisfaction with its performance has been variable so far. It still needs improvements in different domains, preparation, investment in digital technology, assuring equal affordability to all applicants, development of standard validated virtual assessment tools, and continuous feedback and assessment of its performance. The current results of our first Saudi virtual interviewing experience have been encouraging, and they will be an aid for organizers of future events to consider areas of improvement. Future research that compares both virtual and physical interviews with comparison groups or case-control studies would explore the full potential and the pros and cons of each interview style.

## 9. Conclusions

This study shows encouraging results pertaining to virtual interviews as an established and acceptable tool for medical residency matching and the need for ongoing improvement and assessment. Identified areas that need improvement include internet connection and lack of pre-interview demonstration videos. Further research could explore how coping with modern technology trends can promote the quality and efficacy of residency training programs virtual interviews.

## Figures and Tables

**Table 1:** Baseline characteristics of the faculty members.

	Frequency	Percentage
**Years of practice as a consultant/faculty member**		
Less than 5 years	52	30.1
5–10 years	52	30.1
More than 10 years	69	39.9
**Prior to this video interview, did you have prior experience with this tool?**		
No, my first time using video conferencing	59	34.1
Yes, I used video conferencing before, but first time using it for residency interview	109	63
Yes, I used video conferencing before, including for residency interview	5	2.9
**The video conferencing electronic device used**		
PC (laptop)	104	60.1
PC (desktop)	19	11
Mobile	50	28.9

**Table 2:** Faculty members’ indicators of satisfaction with virtual interviews performance.

Indicator	Frequency	Percentage
**Prior to the event, how much of the information that you needed did you get?**		
All of the information	63	36.4
Most of the information	88	50.9
Some of the information	18	10.4
A little of the information	4	2.3
**How organized was the event?**		
Extremely organized	74	42.8
Very organized	79	45.7
Somewhat organized	18	10.4
Not so organized	2	1.2
**Was the event length too long, too short, or about right?**		
Much too long	3	1.7
Too long	12	6.9
About right	150	86.7
Too short	8	4.6
**The interview allowed me to accurately reach an impression of the applicant’s personality.**		
Strongly disagree	15	8.7
Disagree	14	8.1
Neither agree or disagree	31	17.9
Agree	93	53.8
Strongly agree	20	11.6
**My questions for the candidates were answered.**		
Strongly disagree	7	4
Disagree	2	1.2
Neither agree or disagree	9	5.2
Agree	97	56.1
Strongly agree	58	33.5
**I felt comfortable ranking the candidates based on the virtual interviews.**		
Strongly disagree	13	7.5
Disagree	12	6.9
Neither agree or disagree	21	12.1
Agree	80	46.2
Strongly agree	47	27.2
**Indicator**		**Mean, (SD)**
How stressed were you during the video interview?		2.33/5 (1.1)
Overall, how would you rate the event?		4.16/5 (0.89)
How likely is it that you would recommend virtual interviews to a colleague?		8.37/10 (1.50)

## Data Availability

All the data in this study will be made available upon reasonable request upon directly contacting Mohamad-Hani Temsah at mtemsah@ksu.edu.sa.

## Abbreviations

COVID-19	coronavirus disease 2019
Virtual interview	electronic interview video-conferencing
SCFHS	Saudi Commission for Health Specialties
WHO	World Health Organization

## Appendix A

**Residency Application Feedback March 2020 cycle**

Dear Colleague,

Thanks for your valuable participation in the Residency Application with SCFHS in March 2020.

We appreciate it if you can provide us your feedback on your video-conferencing in these interviews.

This is IRB approved proposal, that will also help us to optimize the video-conferencing for residents in the future. Continuing this survey is a consent from you to voluntary participate in this research.

This is an IRB-approved survey that will not be linked to you, and your voluntary participation can help us to further improve the process in the future.

**Thanks and Be Safe!**

Dr. Hani Temsah mtemsah@ksu.edu.sa

Dr. Fadiah AlKhattabi fkhatabi@kfshrc.edu.sa

Dr. Basim Alsaywid drbasim@yahoo.com SCFHS Research Team

1. To start the 5 minutes survey, please choose:

 I took part in the residency interviews of SCFHS residency application in 2020 and I agree to participate I do not agree to participate

Demographics

2. You are:

 Faculty member  Resident applicant  Chief resident  Coordinator

3. Prior to this Video Interview, did you have prior experience in this tool?

 No, my first time to use video conferencing

 Yes I used video conferencing before, but first time to use it for residency interview

Yes I used video conferencing before, including for residency interview

4. The video-conferencing tool you used:

 PC (laptop)  PC (Desk top)  Mobile

5. What is your gender?

 Female    Male

6. Your residency application:

 Pediatrics  Family medicine  Internal medicine Other (please specify)  Surgery Emergency medicine Psychiatr

Faculty page:

7. How much did you find the video interview, as compared to previous traditional face-to-face interviews:

Strongly disagree  Disagree  Neither agree or disagree  Agree  Strongly agree

My questions for the candidates were answered

Strongly disagree  Disagree  Neither agree or disagree  Agree  Strongly agree

8. Which video conferencing tool did you use?

 All through Face Time

 Mostly Face Time, Few Zoom

 Almost equal: Face Time and Zoom Other (please specify)

 Mostly Zoom, Few Face Time All through Zoom

9. What factor(s) made you prefer one video conferencing tool over the other? (Please choose all that apply)

My own personal preference (example: previous experience with Zoom or Face Time)

The candidates preference

The ease of use (user-friendly) of the App

My colleague’s advice

My mobile operating system (Apple versus Android-based)

Other (please specify)

10. Number of your academic experience as consultant:

 less than 5 years  5–10 years  more than 10 years

Applicant’s page:

11. Which video tool did you use in this interview:

 Face Time  Zoom  Other (please specify)

12. How much did you find the video interview, as compared to previous traditional face-to-face interviews:

Strongly disagree  Disagree  Neither agree or disagree  Agree  Strongly agree

My questions about this residency program were answered adequately by the applicant

Strongly disagree  Disagree  Neither agree or disagree  Agree  Strongly agree

13. What factor(s) made you prefer one video conferencing tool over the other? (Please choose all that apply)

My own personal preference (example: previous experience with Zoom or Face Time)

The ease of use (user-friendly) of the App The committee preference

My colleague’s advice

My mobile operating system (Apple versus Android-based)

Other (please specify)

14. Approximately how much do you think this video interview (as compared to coming for face-to-face interview) saved you money in total (example: travel expenses, transportation, hotel accommodations...etc)

 Less than 100 SR  100-500 SR More than 500 SR

Chief Resident or Coordinator page:

15. How much did you find the video interview, as compared to previous traditional face-to-face interviews:

Disagree  Disagree  Neither agree or  disagree  Agree  Strongly agree

My questions for the candidates were answered

Disagree  Disagree  Neither agree or  disagree  Agree  Strongly agree

16. Which video conferencing tool did you use?

 All through Face Time

 Mostly Face Time, Few Zoom

 Almost equal: Face Time and Zoom Other (please specify)

 Mostly Zoom, Few Face Time All through Zoom

17. What factor(s) made you prefer one video conferencing tool over the other? (Please choose all that apply)

My own personal preference (example: previous experience with Zoom or Face Time)

The candidates preference The committee preference

The ease of use (user-friendly) of the App My colleague’s advice

My mobile operating system (Apple versus Android-based)

Other (please specify)

Satisfaction with the interview process:

18. How likely is it that you would recommend the video conferencing interviews to a friend or colleague?

NOT AT ALL LIKELY                    EXTREMELY LIKELY

0          1  2  3          4  5  6          7  8  9          10

19. Currently with the COVID19 Pandemic: How do you find Video Interviews as compared to face-to-face interviews?

 Video Interviews are preferable      Equally preferable      Face-to-face interview are preferable

20. How much do you agree with the following statements regarding this video interview:

Disagree  Disagree  Neither agree or  disagree  Agree  Strongly agreeVideo conferencing interviews decreased the costs for the candidates

21. Overall, how would you rate the event?

Excellent  Very good Good  Fair  Poor

22. What factors improved your video conferencing interview? (please choose all that apply)

Organizers’ communication

WhatsApp group discussion that related specifically to this event

Emails from the organizers

Demo of the video conferencing App before the event

Clear instructions

Free application (Zoom or Face Time) fast internet speed

Other (please specify)

23. What factors negatively affected your video conferencing interview? (please choose all that apply) WhatsApp group discussion that related specifically to this event

 Not receiving the emails from the organizers

 Not having Demo of the video conferencing App before the event

Unclear instructions

 Videoconferencing tool / Application-related (for example: unfamiliar with Zoom or Face Time)

 Slow or interrupted internet speed

Other (please specify)

24. How organized was the event?

 Extremely organized  Very organized  Somewhat organized  Not so organized  Not at all organized

25. Prior to the event, how much of the information that you needed did you get?All of the information  Most of the information  Some of the information   A little of the information  None of the information

26. Was the event length too long too short or about right?

 Much too long  Too long  About right  Too short Much  too short

27. How much do you rate satisfaction with your video-conferencing in regards to:

Extremely satisfied  Extremely unsatisfied   Unsatisfied  satisfied neither Satisfied  nor unsatisfied

. Voice quality

. Your time management flexibility

. Battery/power supply issues

. Your place (office/hospital/home) flexibility

28. Is there anything else you’d like to share about the event?

29. Have you experienced any of the following symptoms immediately after your online video-interview experience?

Never  Almost never  Sometimes  Fairly Often Very Often

. In the last month, how often have you felt that  you were unable to control the important things in your life?

. In the last month, how often have you felt confident about your ability to handle your personal problems?

. In the last month, how often have you found that you could not cope with all the things that you had to do?

. In the last month, how often have you felt that you were on top of things?

. In the last month, how often have you felt difficulties were piling up so high that you could not overcome them?

If you want to receive email updates on this research, please provide your email (it will not be linked with your answers).
